# An Unusual Presentation of Iron-Deficiency Anemia: An Autobiographical Case Report

**DOI:** 10.7759/cureus.20442

**Published:** 2021-12-15

**Authors:** Andrew Kobets, Kseniya Kobets

**Affiliations:** 1 Neurological Surgery, Albert Einstein College of Medicine, New York, USA; 2 Dermatology, Albert Einstein College of Medicine, New York, USA

**Keywords:** autobiographical case report, pica, iron-deficiency, anemia, gerd, gastric reflux, proton pump inhibitor

## Abstract

Gastroesophageal reflux disease (GERD) is one of the most common gastrointestinal disorders and the use of proton-pump inhibitors has become the mainstay of treatment for many patients. While these are the most effective medications for the management of GERD, there are several side effects that patients may experience with their use. This autobiographical case report describes the development of iron-deficiency anemia (IdA) with chronic use of omeprazole. The patient was a 35-year-old male with a history of essential hypertension and GERD who was taking omeprazole 40 mg daily for 3 years for the management of reflux symptoms. He developed some mild exercise intolerance and began noticing an affinity for unusual smells, including gasoline and dust, which prompted an evaluation. Lab work demonstrated IdA to 8.3 g/dl, which was not corrected by oral iron supplementation. Sources of gastrointestinal bleeding, *Helicobacter pylori* infection, and other hypersecretion syndromes were ruled out. IV iron response was transient and only after 8 months of discontinuation of omeprazole did the anemia correct on its own. Omeprazole has increasingly become recognized as a cause of IdA, but only three clinical case reports have been documented in the literature. At least two mechanisms may be involved, and the discontinuation of omeprazole may correct the anemia in 2 months in mild cases, but up to 8 months in more severe cases. The presence of an abnormal propensity for unusual smells, similar to pica as seen in other IdAs, was a unique feature of this case and should prompt evaluation.

## Introduction

Gastroesophageal reflux disease or GERD is one of the most common gastrointestinal disorders with a prevalence between 18 and 27 percent in the United States [1]. It can cause a significant degree of morbidity for patients and chronic symptomatology can result in serious injury to the esophagus. While the exact etiology of the disorder is unknown, it is associated with abnormalities of esophageal motility, lower esophageal sphincter function, and delayed gastric emptying which result in gastric acid reflux injuring the esophagus. Several different medications have been developed over the last few decades for the management of symptoms of GERD in patients for whom lifestyle modification is unsuccessful.

Of the available medications, proton-pump inhibitors (PPIs) have been shown to be most effective for symptoms control in patients with GERD [2]. Since the discovery of omeprazole in 1989, it has become one of the most widely prescribed and effective drugs for symptomatic management [3]. While patients have excellent immediate response with this medication, growing concerns are arising with its long-term use. Rare but serious adverse events include acid hypersecretion, osteoporotic fractures, interstitial nephritis, chronic kidney disease, infection, and nutritional deficiencies, including vitamin B12, magnesium, and iron.

This report is an autobiographical case report in which chronic omeprazole use for the management of GERD resulted in a unique set of symptoms, which prompted medical evaluation that confirmed iron-deficiency anemia (IdA). During work-up, an independent literature review identified two case reports which suggested that omeprazole use could cause the anemia experienced, and transition to another agent corrected the deficiency. This is the first description of this unique constellation of symptoms suggesting IdA, and one of three reports describing anemia induced by omeprazole use described in the literature.

## Case presentation

The patient was a 35-year-old neurosurgical fellow with a medical history significant for essential hypertension and GERD, concurrently with a BMI of 31. The patient had a history of use of an angiotensin-converting enzyme inhibitor for his hypertension but was transitioned to an angiotensin receptor blocker due to a cough with the former medication. For management of the GERD, the patient was initially treating symptoms intermittently with calcium carbonate tablets alone for several years, but for the last 3 years had been taking omeprazole daily, starting with a 20 mg dose, which was escalated to 40 mg over the course of the first year of use. The patient underwent endoscopy after starting omeprazole, which demonstrated esophagitis and a mild hiatal hernia. Lab work was negative for *Helicobacter pylori* infection and no other cause for the GERD was identified. The patient had been managed well on this medication with minimal symptoms but started developing an unusual propensity for certain sharp smells, including gasoline, paint thinner, and dust, which he described as “smell craving,” after more than 2 years of use. He felt this was rather strange and described it as akin to a feeling a pica exhibited by some patients who have cravings to eat inedible materials in states of iron deficiency. Coupled with this feeling, the patient felt a decreased exercise tolerance to the point where after two flights of stairs he felt out of breath. This was a period of heavy clinical workload in his life, and he was unable to exercise as he had at previous points in his life. His only other symptom was the propensity for craving salty foods, particularly ramen noodles.

He was seen at the occupational health center where a complete blood count (CBC) demonstrated a hemoglobin of 8.4 g/dl and a mean corpuscular volume (MCV) of 76 fl, consistent with IdA. A concern for a gastrointestinal bleed was present despite not having any additional symptomatology, and a repeat endoscopy, performed 2 years after the first, demonstrated mild esophagitis, a moderate hiatal hernia, and a few Cameron lesions. A recommendation was made to see a surgeon for a Nissen fundoplication; however, the patient did not elect to undergo surgery at that time. There was a concern that the Cameron lesions were contributing to a slow hemorrhage from these sites, but he experienced no supportive symptomatology, and they were not hemorrhagic. Additionally, his GERD symptoms were well controlled at that time. He remained otherwise asymptomatic, and while he tried to lose weight, the significantly limited exercise tolerance was restrictive.

He investigated the use of long-term PPIs and the development of IdAs given the failure of his work-up to identify another cause and found a few case reports of patients on chronic PPIs who developed IdAs on chronic omeprazole. He read that they recovered after transition to other GERD medications. He tried transitioning off omeprazole at this time to famotidine; however, his symptoms were poorly controlled, and he had to return to omeprazole in order to control the persistent pain he experienced from poor acid control. At this point, he completed his fellowship, moved back to his family, and began work as an Assistant Professor of Neurosurgery. Throughout this time, he remained essentially asymptomatic from his IdA, with good energy levels, persistent feelings of smell craving, and a desire to eat salty ramen noodles. He set an appointment with a new primary care physician who referred him to a hematologist who sent a repeat CBC, which demonstrated a persistently low hemoglobin of 8.2 g/dl with an MCV of 66 fl. White blood cells were normal, as were a lipid panel and endocrine studies. Iron studies demonstrated an iron level of 15 µg/dl, ferritin of 4 ng/ml, and a total iron-binding capacity (TIBC) of 406 µg/dl. Vitamin levels were all within normal limits. The patient was started on oral iron supplementation and, despite his best efforts, was unable to persistently take it due to the dyspepsia he would experience with each administration. Lab studies were repeated the following month and were similar. A decision was then made to give the patient IV iron which he tolerated well. He concurrently lost 10 pounds and was able to transition himself successfully to 80 mg of famotidine daily. The patient underwent a colonoscopy and repeat capsule endoscopy at this time as well without any significant findings or bleeding sources identified.

One month after IV iron, the patient’s hemoglobin improved to 11.9 g/dl and MCV to 79.7 fl with some improvement in exercise tolerance and a loss of his smell cravings. Iron studies improved to an iron level of 58 µg/dl, TIBC of 305 µg/dl, and ferritin of 264 ng/ml. The patient again had repeat lab work 1 month after this, but iron levels began to fall to 33 µg/dl and 25 µg/dl two weeks later, with ferritin going back down to 45 ng/ml and 7 ng/ml after a repeat study. Hemoglobin was slightly lowered to 11.3 g/dl.

One month later, 3 months after his first injection, the patient received a second IV iron injection and his iron levels responded again to 64 µg/dl, 2 weeks after, but then slightly came down to 54 µg/dl at an additional 1-month follow-up. Hemoglobin was 11.8 g/dl 2 weeks after the injection and came up to 12.5 g/dl after an additional 1 month. At this point, the patient had been transitioned to famotidine for 6 months.

The improvement of all symptomatology remained persistent, and labs were again repeated 2 months later, nearly 4 months after his last IV iron injection and 8 months after transitioning off omeprazole. On this follow-up, hemoglobin improved to 14 g/dl, MCV to 88 fl, iron to 62 µg/dl, and ferritin to 65 ng/ml. Typically, the patient experienced falling iron levels 2 months after IV iron but presently his counts remained stable 4 months after. He did not continue his oral iron and did not have any significant changes in his diet, with all other lab work and vitamin levels remaining stable. He continued to remain asymptomatic with complete regulation of his GERD symptoms on famotidine.

## Discussion

Omeprazole is one of the most commonly prescribed PPIs, and other than acid-related diseases, it is useful for the treatment of several other conditions including *H. pylori* infection, gastric cancer, viral infections, and eosinophilic esophagitis [3]. In addition to its effect on other medications metabolized by the cytochrome P450 system, long-term use of omeprazole has been associated with a number of detrimental effects, including nutritional deficiencies from malabsorption of vitamins and minerals, including B12, magnesium, and iron.

Gastric acid plays an important role in the absorption of iron, specifically of the nonheme type derived from fruits, vegetables, grains, and nuts, which requires an acidic environment for absorption [4]. Gastric acid is vital for releasing iron from ligands in foods and solubilizing ferric iron and converting it to its bioavailable form as ferrous iron [5]. It has been speculated that inhibition of gastric acid secretion may lead to a reduction in iron absorption, yet limited data have been compiled over the last few decades to demonstrate the limitation of iron absorption from PPI use. In fact, studies of short-duration omeprazole use have failed to demonstrate reduced iron absorption in iron-replete healthy individuals on a normal diet [4]. This inhibitory iron effect was only experimentally demonstrated in patients who were iron depleted to start, further complicating the true effect of PPIs on iron absorption. 

The maximal effect of omeprazole’s inhibition of gastric acid secretion occurs only after 4 or more days of daily dosing, and it is possible that omeprazole’s effect on the body’s iron-absorbing system may take time to occur. This would coincide with the finding that there is little effect on iron absorption after acute PPIs' use, but rather that iron depletion occurs in patients on chronic therapy, as the effects of PPIs on gastric acid accumulate over time. Few reports of patients on chronic omeprazole who develop IdAs have established a foundation for our demonstration of this effect [6,7]. In fact, they have provided insight into the author’s own management of his anemia.

Iron has additionally been demonstrated to be absorbed through the small intestine via the protein ferroportin, an iron transporter regulated by hepcidin. Hepcidin degrades ferroportin and when hepcidin levels increase, the absorption of iron through the small intestine is decreased. Experimentally, omeprazole has been shown to upregulate hepcidin levels and downregulate ferroportin, providing an additional mechanism through which omeprazole could inhibit iron absorption in certain individuals [8].

To date, there are no common practices to treat iron-deficient patients who are concurrently taking PPIs any differently and it is not common knowledge that PPIs should be suspected in patients without other causes of anemia [9]. In fact, I and many other patients tend to undergo extensive work-up, including invasive procedures, to investigate for more ominous causes of anemia, when a simple transition off omeprazole may suffice. 

For management of PPI-induced IdAs, there may be evidence for the limited efficacy of oral iron in these patients, especially when oral supplementation may worsen gastric discomfort, symptoms for which PPI use was prescribed for in the first place. While patient may be trialed with an IV iron injection, it should be noted that there is only a transient effect of this injection to improve the iron values if the root cause of the deficiency is not identified. Additionally, some diagnostic merit can be attributed to the administration of IV iron, as a hemorrhagic causes for the anemia could be distinguished from a malabsorptive cause by evaluating the patient’s response to this therapy.

The presence of craving of smell is an interesting nuance of this case, and given the patient’s awareness of the pica phenomenon, it is interesting that this link was made to suggest the possibility of iron deficiency prior to a confirmed diagnosis. Pica is the persistent, compulsive craving for and the ingestion of substances usually considered inedible discordant with cultural practices and continues beyond the normal developmental age of indiscriminate ingestion, beyond a period of 1 month [10]. There is an unknown causal link between pica and iron deficiency, but this correlation has been well documented. Patients with these cravings can be highly selective, as was the patient in this case who craved very particular type of smells including gasoline and other musty smells, particularly those not attributable to foods or other normally eaten items. It is interesting to posit that these smell cravings could be a form of pica in a patient unwilling to each inedible material. The indication of possible iron deficiency in an individual aware of this phenomenon is interesting.

In this case, transition to famotidine allowed for the regulation of GERD symptomatology via twice-daily dosing. Cessation of omeprazole resulted in correction of anemia after 8 months. In the other reports, a 49-year-old male had correction of his anemia after 2 months of cessation (although his nadir was 12 g/dl), a 51-year-old female had correction after 2 months (with also a nadir at 12 g/dl), and the final patient, an 81-year-old woman, had correction of her hemoglobin from 9.3 g/dl to 11.8 g/dl after 2 months. The current patient demonstrated the lowest hemoglobin count reported without symptoms of fatigue, despite decreased exercise tolerance. Potentially, his training in a stressful residency afforded him the resilience to manage without feeling fatigued; however, this symptom may in fact be useful as it compels others to seek medical care sooner. In this case, it was, however, his nose that led him to his diagnosis.

## Conclusions

Omeprazole is one of the most commonly prescribed PPIs for the management of GERD and its impact upon vitamin and mineral absorption, specifically iron, is understated in the literature. While a rare phenomenon, patient experiencing fatigue, lethargy, or pica-like symptoms such as smell cravings should be evaluated for a possible link between their PPIs' use and their anemia. Oral supplementation may have some benefit to correct iron levels, and while more effective, IV iron administration is only temporarily efficacious. The transition to another medication for GERD management, in addition to lifestyle modification, may result in improvement of anemia in 2-8 months. The degree of severity of the anemia may impact the time needed for correction.
